# Physical fitness and anthropometric characteristics among adolescents living in urban or rural areas of Kosovo

**DOI:** 10.1186/s12889-017-4727-4

**Published:** 2017-09-16

**Authors:** Faton Tishukaj, Ismet Shalaj, Masar Gjaka, Besim Ademi, Rrustem Ahmetxhekaj, Norbert Bachl, Harald Tschan, Barbara Wessner

**Affiliations:** 1 Centre for Sport Science and University Sports, University of Vienna, Auf der Schmelz 6, 1150 Vienna, Austria; 2 Department of Human Movement and Sport Sciences, University of Rome “Foro Italico”, Piazza Lauro De Bosis 15, 00135 Rome, Italy; 3 Austrian Institute of Sports Medicine, Auf der Schmelz 6, 1150 Vienna, Austria; 4 Research Platform Active Ageing, University of Vienna, Althanstraße 14, 1090 Vienna, Austria

**Keywords:** Physical fitness, Body composition, Urban, Rural, Overweight, Thinness

## Abstract

**Background:**

High physical fitness levels in childhood and adolescence are associated with positive health-related outcomes later in life. Albeit many researchers investigated rural-urban differences in physical fitness, the outcomes of these studies are inconsistent and data on Kosovo are widely missing. Thus, this study aims to examine anthropometric and physical fitness parameters in 14 to 15 year old Kosovan adolescents living in rural and urban areas.

**Methods:**

Two schools from Pristina (mostly urban population) and two schools in the surrounding villages of the district of Deçan (mostly rural population) were selected. Anthropometric and physical fitness parameters were determined from a total of 354 adolescents (216 urban: 14.5 ± 0.4 years, 138 rural: age 14.5 ± 0.4 years) who volunteered to participate in this cross-sectional study performed in 2013/14.

**Results:**

The prevalence of overweight and obesity was 18.9% in girls and 28.2% in males and excess body fat was detected in 18.2% of the girls and 15.9% of the boys with no differences between rural and urban adolescents. Rural adolescents performed slightly better in relative handgrip strength (+4.7%, *p* = 0.032) and running speed (10 m: +2.2%, *p* = 0.012; 20 m: +1.9%, *p* = 0.035), but no other differences were detected in standing long jump, counter movement jump, cardiorespiratory fitness and sit and reach test. A multinomial logistic regression analysis revealed that being a female was associated with a lower relative risk for overweight (RR = 0.11, 95% CI: 0.03–0.34, *p* < 0.001) and high body fat content (RR = 0.20, 95% CI: 0.05–0.56, *p* < 0.001). In addition, higher handgrip strength, longer sprinting time and lower aerobic fitness were associated with a higher relative risk for overweight and excess body fat. In contrast, lower handgrip strength increased the risk for experiencing thinness (RR = 0.92, 95% CI: 0.89–0.96, p < 0.001).

**Conclusions:**

It could be shown that there is a high prevalence of overweight and obesity, especially in 14 to 15 year old boys in Kosovo which does not differ between rural and urban areas. Worse physical performance is associated with a higher risk for overweight and obesity highlighting the importance for interventions in this area and for starting longitudinal observations of a secular trend.

**Electronic supplementary material:**

The online version of this article (10.1186/s12889-017-4727-4) contains supplementary material, which is available to authorized users.

## Background

Evidence suggests that the development of health- and skill-related components of physical fitness early in life are of critical importance for sustainable physical fitness and health outcomes later on [1, 2]. Adolescence is a critical period in this respect with many important physiological and behavioral changes including alterations in body composition and changes in fitness attitudes, physical activity, sedentary behavior and dietary habits [3]. Therefore, country-specific epidemiological data about the prevalence of over- and underweight, body composition and health-related fitness components in adolescents such as cardiorespiratory fitness or muscular fitness are important to design efficient public health strategies and for the development of suitable physical activity interventions.

The Republic of Kosovo represents a post-conflict country in the Western Balkans with the youngest population in Europe and Europe’s highest rate of unemployment. Based on a recent report published by the United Nations, about one fifth of the population is extremely poor, with less than 0.94 €/day per consumption for basic nutritional needs [4]. Additionally, opportunities for leisure and sport activities are very limited and sport facilities and equipment are widely missing [5]. This creates a concern for public health as it has been shown that a lower socio-economic status in childhood is associated with reductions in physical capability levels and a poor health status in adulthood [6, 7].

In addition to the general socio-economic situation within a certain country, differences between rural and urban areas have been highlighted as living in areas with a different population density might affect eating habits [8, 9], access to sport facilities [10, 11], opportunities for physical activity [12] and as a consequence physical fitness and body composition. However, research focusing on physical fitness and physical activity comparing urban and rural youth led to inconsistent results [13–18]. Some authors have suggested that environmental and living conditions in the urban setting are related to a higher level of physical activity and fitness [19, 20], but at the same time an urban environment can be associated with safety concerns [21, 22], a higher usage of computer games [23] and easier access to fast-food restaurants [24, 25] leading to an unhealthy lifestyle.

Health-related physical fitness is highly influenced by anthropometric characteristics such as BMI and body composition. Studies from neighboring countries in the Western Balkans demonstrate that the prevalence of overweight and obesity is rising with negative consequences on health-related fitness parameters [26–30], but data for Kosovo are widely missing. Therefore, the current study aimed to provide first data on health- and skill-related fitness components for Kosovan 14- to 15-year old boys and girls born immediately after the Kosovo conflict that lasted from 1998 to 1999. In addition, the influence of living area (rural versus urban) is analyzed.

## Methods

### Subjects

Participants were recruited from two schools in Pristina, Kosovo (mostly urban population) and two schools in the surrounding villages of the district of Deçan, Kosovo (mostly rural population). The participating schools were chosen as being broadly representative of schools within the capital city and the rural area, respectively, but the final selection of schools was based on the possibility to allow comparable fitness testing in appropriate sports facilities.

The study was approved by the Ethics Committee of the University Clinical Centre of Kosovo (Ethics decision no 6616) and all procedures were performed in accordance with the ethical standards of the Helsinki Declaration as revised in 2013 [31]. Additional authorization was provided by school principals/administrators. Written informed consent was obtained from pupils and parents following a detailed explanation of the testing procedures. All participants were free from any injury or illness that prohibited activity or could have affected the physical fitness measurements.

### Determination of rural and urban areas of living

**Fig. 1:**
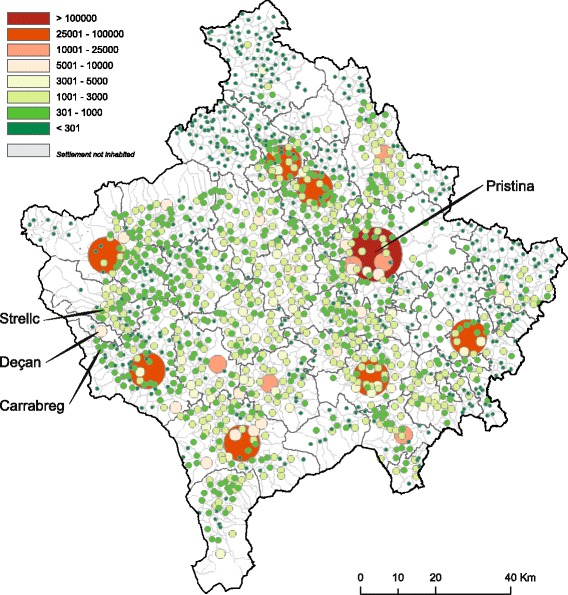
Population distribution in Kosovo. Number of persons by settlement. Adapted from [86]. Permission for use has been obtained from the Kosovo Agency of Statistics

No general consensus exists concerning a definition of residence area in terms of urban and rural area. However, the area of residence for the current study was determined based on data provided by the Kosovo Agency of Statistics [32]. Therefore, an urbanized geographical area - defined at settlement level - is characterized by higher population density and vast human features in comparison to surrounding areas. A rural area is characterized by lower population density, and a higher devotion of the land to agriculture in comparison to surrounding areas. Finally, a specific area is defined as urban or rural by administrative decision of the municipality. Based on the last Kosovo census, 62% of the Kosovan population live in rural areas (urban: 661,586 vs rural: 1,078,239). However, the urban population living in the municipality of Pristina significantly outreaches the rural one [urban: 161,751 (81.3%) vs rural: 37,146 (18.7%)] whilst in the municipality of Deçan, only a small proportion of the population within the municipality lives in an urban area [urban: 3803 (9.5%) vs rural: 36,216 (90.5%)] making these two municipalities a good choice for investigating differences between urban and rural residents (Fig. 1).

### Anthropometric measurements

The structure of data collection was organized at several stations with all measurements performed uniformly concerning the testing order by the same research group during the entire data collection period (November, 2013 – December, 2014). Anthropometric measurements were carried out in the sports hall prior to physical fitness testing according to International Standards for Anthropometric Assessment [33]. Participant’s height was measured to the nearest 0.5 cm using the stretch stature method with a portable stadiometer (Seca, Hamburg, Germany) and body mass was measured to the nearest 0.1 kg using a digital scale (Seca, Hamburg, Germany) with participants dressed in light sports clothing, without shoes.

Body mass index (BMI) was determined from measures of height and body mass using the accepted method (BMI = body mass divided by the square of body height expressed in units kg.m^−2^), and participants were classified as thin, normal weight, overweight and obese using age- and gender-specific cut-off points presented by Cole et al. [34, 35]. In the final analyses overweight and obese students were combined.

Waist circumference (at the level of the narrowest point between the lower costal border and the iliac crest) was measured using an ergonomic circumference tape (Seca 201, Hamburg, Germany). Skinfolds were measured on the right side of the body by an experienced tester, at four anatomical sites (biceps brachii, triceps brachii, the subscapular site and the iliac crest skinfold site) using a skinfold caliper (Harpenden, UK). Each skinfold thickness was measured twice. When the difference between the first and the second measurement exceeded 2 mm, a third measurement was performed, and the mean value of the two most approximate measurements was used for final analysis. The techniques were applied referring to Durnin and Rahman (1967) [36] for the skinfold sites, whereas body fat percentage was calculated from body density (D_ff_) using the gender-specific equations by Weststrate and Deurenberg (1989) [37], for boys: D_ff_ = 1.0715 + 0.00178 · [age (y) – 2], and for girls: D_ff_ = 1.0750 + 0.00313 · [age (y) – 10], respectively, whereas body fat was finally calculated using the following formula: Body fat = [562–4.2 · (age (y) – 2)] / body density – [525–4.7 · (age (y) – 2)]. A body fat content at or above 25% (boys) or 30% (girls) has been associated with a higher risk for cardiovascular diseases in children and adolescents [38]. Using these cut-points, the pupils were classified as having “normal body fat” or “high body fat”.

### Testing physical fitness

Following the anthropometric and body composition assessments, all subjects underwent a series of field-based physical fitness tests performed on two separate days. The physical-fitness test battery comprised selected tests of the EUROFIT test batteries including six physical subtests, quantifying upper-body strength, lower body strength/power, speed, flexibility and cardiovascular endurance [39]. Isometric handgrip strength assessment, sit-and reach-test and standing long jump were performed on the first day, followed by a second day for assessing countermovement jump, 10 m and 20 m sprinting time and the multi-stage fitness test (MSFT). Before testing standing long jump on day 1 and at the beginning of day 2 each participant performed a standardized dynamic warm-up instructed by the test coordinator. The two testing days were interspersed by three days. Except for MSFT and sit-and-reach test, the remaining physical fitness tests were performed twice with the best result used for final analyses. Details about the physical fitness tests applied for the current research are presented in Table 1.

**Table 1:** Summary of physical fitness test items

Testing order	Fitness test item	Motor ability	Test description	Unit of measurement
Day 1, station 1	Isometric handgrip strength	Upper body strength	The maximal force exerted by squeezing a handgrip dynamometer	(kg)
Day 1, station 2	Sit-and-reach test	Flexibility	Maximal reach while sitting with extended knees	(cm)
Day 1, Station 3	Standing long jump	Lower body power	Maximal horizontal jump distance from a standing position	(cm)
Day 2, station 1	Countermovement jump	Lower body power	Maximal vertical jump distance from a standing position	(cm)
Day 2, station 2	10/20 m sprint	Speed	Minimal time needed on a 10/20 m distance run	(s)
Day 2, station 3	Multistage fitness test	Aerobic endurance	Maximal levels achieved while increasing the running speed	(number of shuttles)

#### Upper body strength

Dynamometric measurement of handgrip strength is a validated testing procedure for children and adolescents allowing comparison for this age-group with normative values and centile curves from other European countries [40–42]. After a brief demonstration and adjustment for hand-size, isometric handgrip strength of both, the dominant and the non-dominant hand was measured using a portable hydraulic dynamometer (Jamar, Warrenville, IL, USA). Participants were instructed to sit with their shoulder adducted and neutrally rotated, elbow flexed at 90 degrees and to squeeze the handle with their maximal strength for at least two seconds. Two trials were allowed with each hand with a resting period of 10 s between the attempts, while changing the testing side alternatively. The highest scores achieved from two trials were used for further analyses which are presented in absolute and relative values (mean maximal force production divided by body mass).

#### Lower body strength/power

Explosive power of lower extremities was measured using a countermovement jump (CMJ), performed on a ground reaction force plate (Leonardo Mechanograph, Galileo Novotec Medical GmbH, Germany). Starting from an upright standing position, participants performed a countermovement action, squatting down to a knee angle of approximately 90 degrees before jumping up vertically as explosively as possible, with hands set at the hips. The measurement was performed twice, calculating vertical jumping heights (cm) with highest test scores used for further analyses [43].

In addition to the CMJ a standing long jump was performed. Participants stood with their feet slightly apart (toes behind a starting line) and were asked to jump forward as explosively as possible, landing with parallel feet and without falling backwards. The distance (cm) was measured from the starting line to the back of the heels of the subjects [43].

#### Speed

The running speed for 10 and 20 m was measured electronically to the nearest 0.01 s, using a wireless timing device (Brower Timing Systems, USA) with subjects starting voluntarily. The subjects stood ~40 cm before the starting line on a standing start position and were instructed to run the assigned distance as fast as possible, without performing fluctuation movements at the start [44].

#### Cardiorespiratory fitness

The MSFT was conducted as students ran back and forth between two lines, spaced 20 m apart, in time with the “beep” sounds from a compact disc (20 m Shuttle Run test CD, Australian Sports Commission). Each successful run of the 20 m distance was counted as a complete shuttle. The initial speed was set at 8.5 km·h^−1^ and the pace progressively increased by 0.5 km·h^−1^ every minute of the test resulting in an increased running velocity. The adolescents were warned once if they did not reach the end line in time. The test was terminated when they could not follow the set pace of the “beeps” for two successive shuttles or when they stopped voluntarily. To estimate the maximal oxygen uptake (VO_2max_) from maximal running speed the following formula was used: VO_2max_ (ml·kg^−1^·min^−1^) = 31.03 + 3.24 · speed (km·h^−1^) – 3.25 · age (y) + 0.15 · [speed (km·h^−1^) · age (y)] [45].

#### Flexibility

Research has shown that the sit-and-reach test is a valid and highly reliable measure for hamstring flexibility [46]. No warm-up or stretching exercises were performed by the participants before test measurement. Adolescents were required to sit on the floor with keeping knees fully extended, and soles of the bare feet positioned flat against a standard sit-and-reach box which was fixed against a wall. Participants were instructed to place one hand on top of the other with palms down and slowly reach forward along the surface of the box as far as possible and holding the position of maximal flexion for approximately two seconds. A standard meter ruler was fixed on the sit-and-reach box, with a 0 cm mark representing the point at which the subject’s fingertips were in line with their toes. The sit-and-reach score was measured recorded in centimeters as the final position of the fingertips on the ruler with scores being negative if the girl/boy could not reach her/his toes, and positive when she/he was able to reach beyond the toes.

### Statistical analyses

Means, standard deviations and frequencies were calculated to describe general, anthropometric and physical fitness variables for the total group and individually for boys and girls as well as for urban and rural residents. The Chi-square test was used to compare frequencies between groups. A two-factorial analysis of variance (ANOVA) was performed to detect main effects of sex and living area as well as potential interactions between the categories on physical performance and anthropometric parameters. Differences between independent groups were determined by applying either an independent t-test or a one-factorial ANOVA followed by Scheffe post hoc analyses to suggest homogenous sub-groups. A multinomial logistic regression analysis was used to assess whether physical fitness, gender or living area predict BMI as well as body fat categories. Normal weight and normal body fat respectively were set as reference groups. Sex and area of living were added as factors and handgrip strength, CMJ, standing long jump, 20 m sprint, multistage fitness test and sit and reach were included in the analyses as covariates. In order to account for a potential clustering of the results on schools, a generalized linear mixed model using the above mentioned parameters as fixed factors and school as a random factor was applied in the adjusted models predicting BMI and body fat categories. As normal weight and body fat represent frequent outcomes (> 10%), the odds ratio (OR) derived from logistic regression overestimates the risk ratio (when it is more than 1) or underestimates the risk ratio (when it is less than 1). Therefore, relative risk (RR) was estimated using the formula provided by Zhang and Yu: RR = OR / [(1-P_0_) + (P_0_ *OR), where P_0_ indicates the incidence of the outcome [47]. Statistical significance was set at *p* < 0.05 and all analyses were performed using IBM SPSS statistics version 22.

## Results

### Descriptive characteristics of the study population

The total study sample represents 1.2% of the total number of students attending the 9th grade all around Kosovo [48]. From initially 438 adolescents (265 urban, 173 rural) who were asked to participate, a sample of 354 pupils (80.1%) volunteered to participate in this cross-sectional study and completed all tests (216 urban (81.5%); age 14.5 ± 0.4 years, and 138 rural (79.9%); age 14.5 ± 0.4 years). Unfortunately, there is no information on anthropometric characteristics and physical performance of those not participating in the study, but there was no obvious difference between the study participants and the non-participating pupils.

The number of male students was slightly but not significantly higher in both cohorts (urban: 54.6% male versus 45.4% female; rural: 55.8% male versus 44.2% female, *p* = 0.913) corresponding approximately to the gender distribution within this age group in Kosovo. The prevalence of experiencing thinness in the total sample was 7.3%, 68.6% for normal weight, 24.0% for overweight and obesity. There was a tendency for a higher proportion of boys being overweight or obese (28.2% of boys versus 18.9% of girls), but this did not reach statistical significance (*p* = 0.120). With respect to body fat content, 60 adolescents (16.9%) had a body fat at or above 25% (boys) or 30% (girls). There was no difference in the proportion of children with high body fat content with respect to gender (15.9% of boys versus 18.2% of girls, *p* = 0.572). When comparing the two classification methods (BMI-based versus body-fat based), it was evident that as many as 25 boys (45.5% within BMI category) and 8 (26.7%) girls were classified as overweight based on BMI, but showed normal body fat. In contrast, only one boy (0.8% within BMI category) and seven girls (6.0%) had a high body fat content though being classified as normal weight based on BMI. Against the background of these discrepancies, it was decided to use both classification methods for the following analyses.

In general, girls were slightly younger (*p* = 0.016), shorter (−4.1%, *p* < 0.001), lighter (−9.7%, *p* < 0.001), had a lower waist circumference (−6.7%, *p* < 0.001), but a higher percentage of body fat (+ 43.6%, *p* < 0.001) and a higher sum of skinfolds (+ 26.6%, *p* < 0.001), while BMI was not different between boys and girls (*p* = 0.719). As expected boys performed better in absolute and relative grip strength tests (+36.0% and 22.0%, p < 0.001), standing long jump (33.2%, *p* < 0.001), CMJ (29.2%, p < 0.001), 10 and 20 m sprinting speed (+12.5% and 15.1%, *p* < 0.001), and MSFT (+80.2%, *p* < 0.001), while their sit and reach results were lower (−20.2%, *p* < 0.001).

### Effect of living area on anthropometrics and motor abilities

Area of living did not have any impact on anthropometric data, such as weight, height, BMI, waist circumference, body fat percentage and the sum of skinfolds (Table 2). In addition, the proportion of being thin, normal weight, overweight or obese was similar in rural and urban areas [*p* = 0.977 (girls), *p* = 0.864 (boys)] as determined by Chi-square test. Similar results were obtained for the fraction of boys and girls with normal or high body fat [*p* = 0.712 (girls), *p* = 0.481 (boys)]. With respect to physical fitness tests, higher relative grip strength (+4.7%, *p* < 0.001) and higher sprinting speed (10 m: +2.2%, *p* = 0.012; 20 m: +1.9%, *p* = 0.035) were detected for adolescents living in rural areas. The outcomes of all the other physical fitness tests were similar between pupils living in either rural or urban areas (*p* > 0.05).

**Table 2:** Sex- and area-related differences in anthropometrics and physical fitness

	Boys		Girls		*p*-values		
	Urban (*n* = 118)	Rural (*n* = 77)	Urban (*n* = 98)	Rural (*n* = 61)	Sex	Area	Sex x Area
Age (years)	14.6 ± 0.4	14.6 ± 0.4	14.5 ± 0.4	14.4 ± 0.3	**0.009**	0.185	0.309
Height (m)	1.71 ± 0.08	1.69 ± 0.07	1.62 ± 0.06	1.62 ± 0.06	**< 0.001**	0.132	0.644
Body mass (kg)	63.1 ± 13.6	62.6 ± 14.2	57.5 ± 11.1	55.5 ± 12.1	**< 0.001**	0.393	0.584
Body mass index (kg^·^m^−2^)	21.5 ± 4.2	21.7 ± 4.1	21.7 ± 3.8	21.0 ± 3.9	0.587	0.601	0.399
Waist circumference (cm)	76.4 ± 9.5	75.7 ± 10.6	71.8 ± 7.9	69.8 ± 9.1	**< 0.001**	0.181	0.516
Sum of skinfolds (mm)	42.5 ± 23.9	43.5 ± 32.3	55.9 ± 23.2	51.8 ± 26.2	**< 0.001**	0.587	0.370
Body fat (%)	18.2 ± 6.2	17.9 ± 7.0	26.5 ± 4.5	25.1 ± 5.4	**< 0.001**	0.187	0.430
Grip strength (kg)	34.7 ± 7.3	36.2 ± 8.1	25.9 ± 4.3	26.0 ± 4.6	**< 0.001**	0.277	0.312
Relative grip strength (kg^·^kg^·^BM^−1^)	0.56 ± 0.09	0.59 ± 0.11	0.46 ± 0.09	0.48 ± 0.08	**< 0.001**	**0.032**	0.518
Standing long jump (cm)	175.6 ± 22.3	170.5 ± 26.7	130.2 ± 18.1	130.5 ± 22.7	**< 0.001**	0.321	0.264
Counter movement jump (cm)	38.6 ± 5.5	37.1 ± 5.9	29.3 ± 4.6	29.5 ± 5.6	**< 0.001**	0.288	0.174
10 m sprint (s)	2.14 ± 0.15	2.08 ± 0.13	2.43 ± 0.17	2.40 ± 0.18	**< 0.001**	**0.012**	0.334
20 m sprint (s)	3.69 ± 0.27	3.61 ± 0.26	4.33 ± 0.30	4.28 ± 0.32	**< 0.001**	**0.035**	0.591
Multistage fitness test (levels)	6.5 ± 1.7	6.3 ± 2.3	3.5 ± 1.1	3.6 ± 1.0	**< 0.001**	0.574	0.462
Multistage fitness test (m)	934 ± 459	919 ± 459	407 ± 179	415 ± 182	**< 0.001**	0.925	0.727
VO_2max_ (ml^·^kg^-1·^min^−1^)	45.0 ± 4.6	44.1 ± 6.3	37.2 ± 2.9	37.3 ± 2.9	**< 0.001**	0.412	0.343
Sit and reach (cm)	20.8 ± 7.3	21.5 ± 6.5	26.7 ± 6.4	26.0 ± 6.5	**< 0.001**	0.989	0.312

Data are expressed as means ± standard deviations; Main and interaction effects between sex and area of living were analyzed by two factorial ANOVA; Statistical significant differences are marked in bold

Abbreviations: *BM* body mass, *VO*_*2max*_ maximal oxygen uptake

### Effects of body mass and body fat content on performance parameters

As summarized in Table 3 weight, BMI, waist circumference sum of skinfolds and body fat differed significantly between thin, normal weight, overweight and obese boys and girls (*p* < 0.001). Thin boys were about 9 cm smaller than normal, overweight and obese boys (*p* < 0.001). While absolute grip strength was highest in overweight and obese boys and girls (*p* < 0.001), relative grip strength was higher in normal weight and thin adolescents (*p* < 0.001). Normal weight boys showed better performances in standing long jump, CMJ, sprinting speed, MSFT and estimated VO_2max_ than both, thin and overweight boys. Interestingly, thin girls performed better in these variables than both, normal weight and overweight girls. Taken together these data clearly show that physical performance is negatively influenced by overweight and obesity in both genders. Similarly, a body fat content of more than 25% (boys) and 30% (girls) was associated with worse outcomes in all physical performance tests with the exception of flexibility (sit and reach) and absolute handgrip strength as shown in Table 4.

**Table 3:** Anthropometric and physical fitness parameters based on BMI categories

	Boys				Girls			
	Low weight (a)	Normal weight (b)	Overweight & Obese (c)	*p*-value	Low weight (a)	Normal weight (b)	Overweight & Obese (c)	*p*-value
N (%)	13 (6.7%)	127 (65.1%)	55 (28.2%)		13 (8.2%)	116 (77.3%)	30 (18.9%)	
Age (years)	14.6 ± 0.4^1^	14.6 ± 0.4^1^	14.6 ± 0.4^1^	0.779	14.5 ± 0.3^1^	14.5 ± 0.4^1^	14.6 ± 0.3^1^	0.258
Height (m)	1.62 ± 0.08^1^	1.71 ± 0.07^2^	1.71 ± 0.07^2^	< 0.001 (ab, ac)^***^	1.64 ± 0.06^1^	1.62 ± 0.05^1^	1.65 ± 0.06^1^	0.069
Body mass (kg)	41.5 ± 6.4^1^	58.3 ± 7.1^2^	78.7 ± 12.0^3^	< 0.001 (ab, ac, bc)^***^	44.2 ± 4.0^1^	53.5 ± 6.0^2^	74.7 ± 11.2^3^	< 0.001 (ab, ac, bc)^***^
Body mass index (kg^·^m^−2^)	15.7 ± 1.2^1^	19.8 ± 1.7^2^	27.0 ± 3.1^3^	< 0.001 (ab, ac, bc)^***^	16.4 ± 0.6^1^	20.4 ± 1.8^2^	27.6 ± 3.6^3^	< 0.001 (ab, ac, bc)^***^
Waist circumference (cm)	63.1 ± 4.2^1^	72.4 ± 4.2^2^	87.8 ± 9.9^3^	< 0.001 (ab, ac, bc)^***^	62.8 ± 4.2^1^	68.7 ± 5.3^2^	83.5 ± 7.4^3^	< 0.001 (ab, ac, bc)^**^
Skinfold sum (mm)	22.4 ± 2.4^1^	31.4 ± 8.7^1^	74.3 ± 33.3^2^	< 0.001 (ac, bc)^***^	29.8 ± 5.3^1^	47.5 ± 12.5^2^	91.3 ± 27.3^3^	< 0.001 (ab, ac, bc)^**^
Body fat (%)	11.5 ± 1.4^1^	15.4 ± 3.4^2^	25.7 ± 6.2^3^	< 0.001 (ab, ac, bc)^**^	19.4 ± 2.2^1^	24.8 ± 3.2^2^	33.0 ± 3.8^3^	< 0.001 (ab, ac, bc)^***^
Grip strength (kg)	26.4 ± 5.9^1^	34.5 ± 7.2^2^	39.1 ± 7.0^3^	<0.001 (ab, ac, bc)^***^	23.1 ± 4.2^1^	25.5 ± 3.9^1^	28.8 ± 5.2^2^	< 0.001 (ac, bc)^***^
Relative grip strength (kg^·^kg BM^−1^)	0.63 ± 0.07^2^	0.59 ± 0.10^2^	0.50 ± 0.09^1^	<0.001 (ac, bc)^***^	0.52 ± 0.07^2^	0.48 ± 0.07^2^	0.39 ± 0.09^1^	<0.001 (ac, bc)^***^
Standing long jump (cm)	171.6 ± 24.3^1^	177.7 ± 22.3^1^	164.6 ± 26.2^1^	0.003 (bc)^**^	145.7 ± 28.5^3^	131.9 ± 18.3^2^	117.3 ± 14.6^1^	< 0.001 (ab, ac, bc)^*^
CMJ (cm)	37.0 ± 6.7^1^	39.0 ± 5.4^1^	35.9 ± 5.6^1^	0.002 (bc)^**^	32.7 ± 4.0^2^	29.9 ± 4.9^2^	26.2 ± 4.6^1^	<0.001 (ac, bc)^**^
10 m sprint (s)	2.11 ± 0.17^1,2^	2.09 ± 0.14^1^	2.19 ± 0.14^2^	< 0.001 (bc)^***^	2.35 ± 0.17^1^	2.42 ± 0.17^1,2^	2.48 ± 0.17^2^	0.070
20 m sprint (s)	3.67 ± 0.34^1,2^	3.59 ± 0.23^1^	3.82 ± 0.27^2^	< 0.001 (bc)^***^	4.18 ± 0.31^1^	4.29 ± 0.30^1,2^	4.47 ± 0.34^2^	0.004 (ac, bc)^*^
MSFT (levels)	6.0 ± 1.7^1^	7.1 ± 1.7^2^	4.9 ± 1.7^1^	< 0.001 (bc)^***^	3.8 ± 1.2^2^	3.7 ± 1.0^1,2^	3.0 ± 1.0^1^	0.008 (bc)^*^
MSFT (m)	837 ± 311^1^	1069 ± 353^2^	624 ± 300^1^	<0.001 (bc)^***^	445 ± 194^1^	429 ± 178^1^	323 ± 156^1^	0.011 (bc)^*^
VO_2max_ (ml·kg^−1^·min^−1^)	43.0 ± 4.7^1^	46.6 ± 4.7^2^	40.6 ± 4.5^1^	< 0.001 (ab, bc)^*^	37.6 ± 3.0^1^	37.6 ± 2.9^1^	35.8 ± 2.8^1^	0.008 (bc)^**^
Sit and reach (cm)	18.1 ± 6.8^1^	20.8 ± 7.0^1^	22.5 ± 6.7^1^	0.095	25.3 ± 7.9^1^	26.2 ± 6.4^1^	28.0 ± 5.8^1^	0.309

Data are expressed as means ± standard deviations; Differences between BMI categories as analyzed by ANOVA followed by Scheffe post hoc analyses; asterisks mark significant differences between BMI categories (^*^ *p* < 0.05,^**^ *p* < 0.01,^***^ *p* < 0.001); superscript numbers denote homogenous subgroups according to Scheffe;

Abbreviations: *BMI* body mass index, *BM* body mass, *CMJ* counter movement jump, *MSFT* multistage fitness test, *VO*_*2max*_ maximal oxygen uptake

**Table 4:** Anthropometric and physical fitness parameters based on body fat categories

	Boys			Girls		
	Normal body fat	High body fat	*p*-value	Normal body fat	High body fat	*p*-value
N (%)	164 (84.1%)	31 (15.9%)		130 (81.8%)	29 (18.2%)	
Age (years)	14.6 ± 0.4	14.6 ± 0.4	0.728	14.5 ± 0.4	14.6 ± 0.4	0.121
Height (m)	1.70 ± 0.08	1.71 ± 0.06	0.700	1.62 ± 0.05	1.65 ± 0.06 ^*^	0.017
Body mass (kg)	59.0 ± 10.0	83.5 ± 13.2 ^***^	< 0.001	52.9 ± 6.7	74.1 ± 12.6 ^***^	< 0.001
Body mass index (kg^·^m^−2^)	20.3 ± 2.8	28.5 ± 3.3 ^***^	< 0.001	20.1 ± 2.3	27.2 ± 4.3 ^***^	< 0.001
Waist circumference (cm)	73.0 ± 6.4	92.9 ± 8.6 ^***^	< 0.001	68.3 ± 5.8	83.2 ± 7.8 ^***^	< 0.001
Skinfold sum (mm)	32.8 ± 10.9	96.2 ± 27.2 ^***^	< 0.001	45.0 ± 11.2	96.1 ± 23.8 ^***^	< 0.001
Body fat (%)	15.8 ± 3.9	30.0 ± 3.6 ^***^	< 0.001	24.2 ± 3.2	33.8 ± 3.0 ^***^	< 0.001
Grip strength (kg)	34.6 ± 7.5	38.8 ± 7.6 ^**^	0.005	25.5 ± 4.2	27.8 ± 4.8 ^**^	0.010
Relative grip strength (kg^·^kg^·^BM^−1^)	0.59 ± 0.09	0.47 ± 0.08 ^***^	< 0.001	0.48 ± 0.07	0.38 ± 0.08 ^***^	< 0.001
Standing long jump (cm)	178.2 ± 22.3	149.2 ± 19.0 ^***^	< 0.001	133.4 ± 19.8	116.5 ± 14.0 ^***^	< 0.001
CMJ (cm)	38.9 ± 5.3	33.5 ± 5.5 ^***^	< 0.001	30.3 ± 4.9	25.5 ± 4.0 ^***^	< 0.001
10 m sprint (s)	2.10 ± 0.14	2.23 ± 0.15 ^***^	< 0.001	2.41 ± 0.17	2.47 ± 0.19	0.080
20 m sprint (s)	3.62 ± 0.25	3.88 ± 0.28 ^***^	< 0.001	4.27 ± 0.30	4.50 ± 0.33 ^***^	< 0.001
MSFT (levels)	6.8 ± 1.8	4.4 ± 1.4 ^***^	< 0.001	3.7 ± 1.1	2.9 ± 0.7 ^***^	< 0.001
MSFT (m)	999 ± 371	553 ± 244 ^***^	< 0.001	436 ± 181	294 ± 116 ^***^	< 0.001
VO_2max_ (ml·kg^−1^·min^−1^)	45.7 ± 5.0	39.2 ± 3.8 ^***^	< 0.001	37.6 ± 2.9	35.4 ± 2.1 ^***^	< 0.001
Sit and reach (cm)	21.0 ± 6.8	21.6 ± 8.0	0.666	26.3 ± 6.5	27.3 ± 6.2	0.426

Data are expressed as means ± standard deviations; Differences between body fat categories as analyzed by unpaired t-test; asterisks mark significant differences between body fat categories (^*^ *p* < 0.05, ^**^ *p* < 0.01, ^***^ *p* < 0.001)

Abbreviations: *BMI* body mass index, *BM* body mass, *CMJ* counter movement jump, *MSFT* multistage fitness test, *VO*_*2max*_ maximal oxygen uptake

**Fig. 2:**
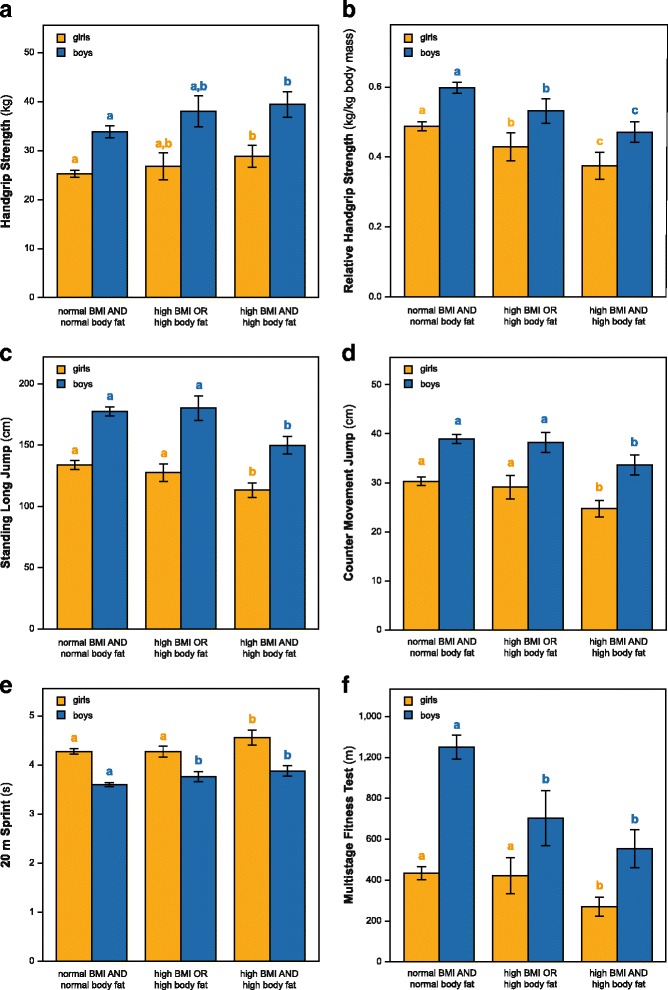
Combined influence of BMI and body fat categories on physical performance. Groups were built based on the combined occurrence of normal (thin & normal weight adolescents) or high BMI (overweight & obese adolescents) and normal or high body fat percentage. **a** Absolute handgrip strength, **b** relative handgrip strength, **c** standing long jump, **d** counter movement jump, **e** 20 m sprint time, and **f** multistage fitness test are shown separately for boys and girls. Bars represent the mean ± 95% CI. Different lower case letters (a, b, c) denote different subgroups within the sex category as determined by one factorial ANOVA followed by Scheffe post-hoc analyses (*p* < 0.05)

As there were 261 adolescents of the total population (73.7%) with normal or low BMI together with normal body fat, 41 adolescents (11.6%) with either a high BMI or a high body fat content, and 52 adolescents (14.7%) with high BMI plus high body fat, it was tested whether there would be a difference in performance between these three groups having either no, one or both “anthropometric risk factors” (Fig. 2). The latter group (high BMI plus high body fat) had a higher absolute handgrip strength (girls: +3.5 kg, boys: +5.6 kg, *p* < 0.01), but a lower performance in standing long jump (girls: −20 cm, boys: −27 cm, *p* < 0.001), CMJ (girls: −5.6 cm, boys: −5.3 cm, *p* < 0.001, *p* < 0.001), 20 m sprint (girls: +0.28 s, boys: +0.28 s, *p* < 0.001), and the multistage fitness test (girls: −164 m, boys: −498 m, p < 0.001). In contrast, the occurrence of just one risk factor (either high BMI or high body fat) did not lead to worse outcomes in handgrip strength (Fig. 2A), standing long jump (Fig. 2C), and CMJ (Fig. 2D) in both genders, and in 20 m sprint (Fig. 2E) and the multistage fitness test (2F) only in female but not in male pupils. Interestingly, relative handgrip strength (absolute handgrip strength divided by body mass) was able to clearly discriminate between the three groups with the worst results in the high BMI plus high body fat group (girls: −0.11 kg/kg body mass, boys: −0.13 kg/kg body mass, *p* < 0.001) (Fig. 2B).

### Determinants of being thin or overweight

In order to model the relationship between BMI categories and several potential predictors (gender, living area, physical fitness parameters) a multinomial logistic regression was performed. The addition of the predictors to a model that contained only the intercept significantly improved the fit between model and data [χ^2^ (16) = 187.7, Nagelkerke R^2^ = 0.517, *p* < 0.001]. Gender [χ^2^ (2) = 29.7, *p* < 0.001], handgrip strength [χ^2^ (2) = 84.5, *p* < 0.001], aerobic fitness as assessed by multistage fitness test [χ^2^ (2) = 34.6, *p* < 0.001], and standing long jump [χ^2^ (2) = 6.4, *p* = 0.044] contributed significantly to the model. Table 5 presents the results of the unadjusted and adjusted multinomial logistic regression. Being a female was associated with a lower risk for being overweight or obese in both models (adjusted model: RR = 0.10, 95% CI: 0.03–0.34, *p* < 0.001). Living in either an urban or rural are did not predict the body mass classification. Higher handgrip strength (adjusted model: RR = 1.06, 95% CI: 1.04–1.08, *p* < 0.001), longer sprinting time (adjusted model: RR = 1.35, 95% CI: 1.02–1.44, *p* = 0.041) and lower aerobic fitness (adjusted model: RR = 0.999, 95% CI: 0.998–0.999, *p* < 0.001) were associated with a higher risk for being overweight or obese. On the other hand, a lower handgrip strength slightly increased the risk of being thin (adjusted model: RR = 0.92, 95% CI: 0.89–0.96, *p* < 0.001).

**Table 5:** Multinomial logistic regression predicting weight categories

	Unadjusted model		Adjusted model			
	Low weight	Overweight & Obese	Low weight	Overweight & Obese	Low weight	Overweight & Obese
	OR (95% CI)	OR (95% CI)	OR (95% CI)	OR (95% CI)	RR (95% CI)	RR (95% CI)
Gender ^a^						
Female	1.095 (0.488–2.458)	0.597 (0.358–0.996)^*^	1.032 (0.210–5.068)	0.037 (0.010–0.137)^***^	1.010 (0.458–1.337)	0.109 (0.031–0.336)^***^
Area of living^b^						
Rural area	1.162 (0.512–2.639)	1.006 (0.606–1.670)	1.038 (0.403–2.675)	0.713 (0.317–1.604)	1.012 (0.683–1.245)	0.888 (0.596–1.134)
Grip strength (kg)	0.868 (0.802–0.940)^***^	1.090 (1.054–1.126)^***^	0.789 (0.710–0.877)^***^	1.229 (1.149–1.314)^***^	0.923 (0.886–0.958)^***^	1.062 (1.042–1.081)^***^
Standing long jump (cm)	1.003 (0.990–1.016)	0.992 (0.983–1.000)^*^	1.027 (0.998–1.058)	0.983 (0.962–1.004)	1.008 (0.999–1.018)	0.995 (0.998–1.001)
CMJ (cm)	1.004 (0.946–1.065)	0.954 (0.919–0.990)^*^	1.076 (0.944–1.227)	0.935 (0.856–1.022)	1.023 (0.982–1.062)	0.979 (0.950–1.007)
20 m sprint (s)	0.989 (0.384–2.548)	1.919 (1.089–3.379)^*^	0.460 (0.036–5.834)	5.591 (1.075–29.077)^*^	0.731 (0.106–1.352)	1.347 (1.022–1.435)^*^
MSFT (m)	0.999 (0.998–1.000)	0.998 (0.997–0.998)^***^	0.998 (0.996–1.000)	0.996 (0.995–0.998)^***^	0.999 (0.999–1.000)	0.999 (0.998–0.999)^***^
Sit and reach (cm)	0.969 (0.917–1.024)	1.020 (0.985–1.057)	0.958 (0.889–1.034)	1.047 (0.997–1.100)	0.986 (0.962–1.010)	1.014 (0.999–1.029)

Normal weight was chosen as reference group for the outcome. ^a^ Reference category is “male”; ^b^ Reference category is “urban area”. In the unadjusted model each variable was tested independently as a predictor of weight categories. In the adjusted model, all the variables were tested in the same model, controlling the effect of each other. In addition, school was included as random factor in order to control for potential cluster effects. RR was estimated from OR using the conversion formula from Zhang and Yu [48]. ^*^ *p* < 0.05, ^***^ *p* < 0.001

Abbreviations: *OR* odds ratio, *RR* relative risk, *CI* confidence interval, *CMJ* counter movement jump, *MSFT* multistage fitness test

### Determinants of body fat content

In order to assess the impact of gender, living area and physical fitness parameters on body fat content a second multinomial logistic regression was performed. The addition of the predictors to a model that contained only the intercept significantly improved the fit between model and data [χ^2^ (8) = 136.8, Nagelkerke R^2^ = 0.536, *p* < 0.001]. Gender [χ^2^ (1) = 21.8, p < 0.001], handgrip strength [χ^2^ (1) = 34.9, p < 0.001], standing long jump [χ^2^ (1) = 23.1, *p* < 0.001], CMJ [χ^2^ (1) = 23.1, *p* = 0.004], and aerobic fitness [χ^2^ (1) = 17.5, p < 0.001] contributed significantly to the model. The detailed results of the unadjusted and adjusted multinomial logistic regression are shown in Table 6. Female sex was associated with a lower risk for having a high body fat content (adjusted model: RR = 0.20, 95% CI: 0.05–0.56, *p* < 0.001), whereas the living area did not influence body composition. Higher handgrip strength (adjusted model: RR = 1.03, 95% CI: 1.02–1.04, *p* < 0.001), but worse results in standing long jump (adjusted model: RR = 0.99, 95% CI: 0.98–0.99, *p* < 0.001), CMJ (adjusted model: RR = 0.97, 95% CI: 0.96–0.99, *p* = 0.007), and aerobic fitness (adjusted model: RR = 0.999, 95% CI: 0.999–1.000, *p* < 0.001) were associated with a higher risk of having a higher body fat percentage.

**Table 6:** Multinomial logistic regression predicting body fat categories

	Unadjusted model	Adjusted model	
	High body fat	High body fat	High body hat
	OR (95% CI)	OR (95% CI)	RR (95% CI)
Gender ^a^			
Female	1.180 (0.677–2.058)	0.040 (0.009–0.176)^***^	0.197 (0.051–0.558)^***^
Area of living^b^			
Rural area	1.243 (0.708–2.180)	0.599 (0.229–1.569)	0.898 (0.637–1.065)
Grip strength (kg)	1.046 (1.011–1.082)^*^	1.212 (1.126–1.303)^***^	1.031 (1.019–1.041)^***^
Standing long jump (cm)	0.969 (0.958–0.980)^***^	0.940 (0.914–0.966)^***^	0.989 (0.984–0.994)^***^
CMJ (cm)	0.879 (0.838–0.921)^***^	0.866 (0.781–0.960)^**^	0.974 (0.955–0.993)^**^
20 m sprint (s)	4.152 (2.146–8.035)^***^	0.572 (0.090–3.612)	0.887 (0.368–1.140)
MSFT (m)	0.997 (0.996–0.998)^***^	0.996 (0.994–0.998)^***^	0.999 (0.999–1.000)^***^
Sit and reach (cm)	1.020 (0.981–1.061)	1.034 (0.980–1.092)	1.006 (0.997–1.014)

Normal body fat (boys <25%, girls <30%) was chosen as reference group for the outcome. ^a^ Reference category is “male”; ^b^ Reference category is “urban area”. In the unadjusted model each variable was tested independently as a predictor of body fat. In the adjusted model, all the variables were tested in the same model, controlling the effect of each other. In addition, school was included as random factor in order to control for potential cluster effects. RR was estimated from OR using the conversion formula from Zhang and Yu [48]. ^*^ *p* < 0.05, ^**^ *p* < 0.001, ^***^ *p* < 0.001

Abbreviations: *OR* odds ratio, *RR* relative risk, *CI* confidence interval, *CMJ* counter movement jump, *MSFT* multistage fitness test

## Discussion

This study provides data regarding health- and skill related fitness components related to rural or urban living area among Kosovan adolescents residing in Pristina and two surrounding villages of Deçan. The results indicate no significant differences in BMI and body fat content between urban and rural adolescents, and only moderate advantages in physical fitness parameters (sprinting time, relative grip strength) for rural boys and girls. While authors from Spain [13], Portugal [15], Greece [49], Taiwan [18] and India [50] reported better physical fitness results achieved by children and adolescents living in rural areas, others from Croatia, Ecuador and Mexico argue that an urban environment seems to be more advantageous [17, 51, 52] showing the difficulty to clearly judge the impact of the place of residence on physical fitness.

A major determinant of physical fitness is of course the level of physical activity [53, 54], which is related to intrapersonal, interpersonal, organizational, environmental, policy as well as socio-economic factors [55, 56]. Although physical activity levels were not measured in this study, it may be speculated that the participation level would be similarly low in rural and urban children. Infrastructure and the built environment such as homes, schools, workplaces and industrial areas, highways, shops, parks and public places have been shown to impact physical activity in both positive and negative ways [57, 58]. A recent review suggests that limited active living built environments in rural communities and unique rural barriers to physical activity arising from travel distance and transportation may exist [59]. In Kosovo, the administrative environment, in which the practice of urban planning and mobility management is embedded, is characterized by a high degree of instability characterized by loose control by the authorities [60]. In this respect it can be assumed that especially the urban environment in Pristina does not facilitate an active lifestyle of adolescents. In addition, safety issues (high crime rate and the presence of loose dogs) as well as air pollution may influence the habits of adolescents themselves but may also cause parental barriers to allow leisure time sports or to actively commute to school [61, 62]. However, after the war, Kosovo has been and still is, subject to substantial population relocations, with people moving due to socio-economic reasons from all parts of Kosovo to the capital city Pristina, which could lead to population interfusion making differences between rural and urban areas less pronounced. The current study provided first data for Kosovan adolescents, but future studies should add further information on specific living conditions, such as type of housing, number of occupants, the availability of sports facilities and the general built environment which would be needed in order to determine the impact of the environment on health- and fitness-related parameters specific for urban and rural parts of Kosovo.

The physical fitness level of Kosovan adolescents as measured in our study is close to the 50th percentile for both boys and girls as compared to a large study combining data from 10 European cities in Austria, Belgium, France, Germany, Greece, Hungary, Italy, Spain and Sweden [63]. A further study comparing 23 European countries revealed that there is a considerable variability in the relative performance on the Eurofit test battery, but in general countries of northern and central Europe outperformed those of western and southern Europe. Kosovo was not included, but Albania, comparable in many aspects to Kosovo, was ranked on 18th place [64].

Besides physical performance, BMI and body composition have been suggested to be included in physical fitness test batteries representing morphological components of health-related fitness as they influence the results of physical performance tests and in addition, BMI and especially body composition are considered as independent indicators of health [65]. In the current study the BMI-based prevalence of overweight and obesity was 24.0% with a trend for being slightly higher in boys (28.2%) than in girls (18.9%). At the same time 7.3% of the adolescents were thin. Another study investigating 1228 Kosovan students aged 15 to 18 years revealed a similar prevalence of 7.0% for thinness, but only 10.4% for overweight with higher values for overweight boys (15.2%) than girls (5.6%) [66]. However, the results reported by the latter study are based on self-reported BMI calculations which have been shown to overestimate height and underestimate weight leading to a lower proportion of overweight subjects [67]. A further aspect that needs to be considered is a potential bias based on the socioeconomic status of the study participants. It has been shown that obesity in children appears to be predominantly a problem of the rich in low- and middle-income countries [68]. Therefore, the proportion of overweight and obese children could be overestimated if the socio-economic status of the study participants and their parents would be higher as compared to the whole population in Kosovo. However, as only public schools without any access restrictions and located in two different areas (rural and urban) were included in the current study, we think that the risk of bias with respect to the socio-economic status is rather small in the current study.

In line with the present study no differences in the prevalence of overweight and obesity between urban and rural areas have been reported for Poland [69] and Croatia [17], but rural adolescents were more likely to be overweight in Greece, Romania and Portugal [49, 70, 71]. It has to be mentioned that most of the studies comparing rural and urban areas contain cross-sectional data not taking into account that changes over time may occur. This has been shown in China, where the prevalence of combined overweight and obesity was significantly higher in urban than in rural children in 1985, 1995 and 2005. However, a rapid increase in the prevalence of combined overweight and obesity occurred in rural areas after 2005 and as a result, the urban-rural disparity was getting narrower with a lack of urban-rural disparity in 2014 [72].

Besides BMI, especially a high body fat content is associated with a higher risk for chronic diseases as shown in 12,279 children and adolescents from the National Health and Nutritional Examination Surveys III and IV [73]. While 24% of the study participants were classified as overweight or obese based on BMI, only 17% showed excess body fat. The calculation of these prevalence data is highly dependent on the used cut-points which are widely accepted for BMI but to a lesser extent for body fat percentage, although some authors have raised concerns about the validity of the BMI as an surrogate indicator of body fatness and health risk in children and adolescents [65, 74]. In the current study a body fat content at or above 25% (boys) or 30% (girls) was considered as border between normal and high body fat as these cut-points have been demonstrated to be associated with a higher cardiovascular disease risk in children and adolescents [38]. It is noteworthy that boys and girls with either high BMI or high body fat performed worse than those having normal BMI or body fat in all weight-bearing tasks such standing long jump, CMJ, sprint and MSFT. Similar results have been obtained very frequently confirming the negative association between higher body mass, body fat and physical fitness [75–77]. As BMI and body fatness comprise slightly different risk factors for poor health status we identified those students having both, a high BMI plus high body fat (14.7%) as suggested by Hung et al. [78]. Interestingly, this subgroup was less fit than the reference group (normal BMI and normal body fat) but also weaker than those having only one anthropometric risk factor (either high BMI or high body fat). Therefore, we suggest to combine BMI and body fat measurements for improving the accuracy of obesity screening in adolescents.

Besides showing a strong influence of gender, the combined analysis of physical performance and weight categories or body composition using multinomial logistic regression analyses revealed higher handgrip strength, longer sprinting time and lower aerobic fitness to be associated with a higher risk for being overweight and obese. At the same time lower handgrip strength was associated with an increased risk of experiencing thinness. Special attention should be paid to absolute handgrip strength which is positively associated with higher body mass and also body fat content. Weak handgrip strength is associated with undernutrition in pediatric patients [79], and a worse metabolic risk profile (systolic and diastolic blood pressure, triglycerides and C-reactive protein) in 10 to 12 year old children [80]. In adults, higher handgrip strength is associated with a better health status, but it has been shown recently that relative handgrip strength might better predict a disproportion between muscle mass and muscle fat that may occur in obese children and adolescents [81]. Unfortunately, it was not possible to measure metabolic risk factors in our study and to relate them to (relative) handgrip strength, but it is highly recommended for further studies as the surveillance of chronic non-communicable risk factors in children born in Lower Middle Income Countries and Developing Countries with simple means would be of utmost relevance for public health authorities in these countries.

### Limitations

It has to be mentioned that the findings of the current study are subject to some limitations. The selection of schools was based on the availability of gymnastic halls which might not be representative for Kosovo, where indoor sports facilities in schools are widely missing. As we aimed to create comparable testing conditions for rural and urban children, the availability of an indoor gym was a prerequisite to conduct the current study. Although the weekly physical education lessons are the same in all schools (with or without gyms) physical fitness could be higher due to better exercise conditions in comparison to other schools in Kosovo [82, 83]. For further studies we highly recommend to assess the availability of sports and recreational facilities as well as other environmental conditions in addition to physical fitness parameters. Furthermore, it would have been interesting to assess the socio-economic status as health behaviors have been shown to rely on income and education [84]. Third, our data are based on a rather narrow age-group (14 to 15 year old), making it difficult to extend our conclusion to younger or older children and adolescents. In this context a recently published study has to be mentioned, which investigated anthropometric characteristics of 352 preschool children (12–59 months old) [85]. Combined efforts should be undertaken to prepare a concerted data base (using standardized methods) in order to get a representative picture of health-related data for Kosovan children and adolescents.

## Conclusions

In conclusion, and within this study’s limitations, we could show that there is a high prevalence of overweight and obesity, especially in 14 to 15 year old boys in Kosovo which does not differ between rural and urban areas. Worse physical performance is associated with a higher risk for overweight and obesity as well as high body fat content highlighting the importance for interventions in this area, but further studies extending the research to other age groups and including environmental and socio-economic factors are needed to get a clear cross-sectional picture providing the basis for representative longitudinal studies. Monitoring changes in health-related data such as physical performance, anthropometric parameters and other risk factors (metabolic risk factors, smoking and alcohol consumption, physical inactivity or similar) over time could be extremely helpful for health professionals and decision makers to observe secular trends and to design interventions and actions specifically suitable for the needs of the geographical region.

## Additional file

Additional file 1: Fitness and anthropometric raw data (XLSX 54 kb)

## Abbreviations

ANOVA: Analysis of Variance
BMI: Body Mass Index
CI: Confidence Interval
CMJ: Countermovement Jump
Dff: Body density
MSFT: Multi-stage Fitness Test
OR: Odds Ratios
VO_2max_: Maximal Oxygen Uptake
